# Cancer-associated mutations in the iron-sulfur domain of *FANCJ* affect G-quadruplex metabolism

**DOI:** 10.1371/journal.pgen.1008740

**Published:** 2020-06-15

**Authors:** Diana C. Odermatt, Wei Ting C. Lee, Sebastian Wild, Stanislaw K. Jozwiakowski, Eli Rothenberg, Kerstin Gari

**Affiliations:** 1 Institute of Molecular Cancer Research, University of Zurich, Zurich, Switzerland; 2 Department of Biochemistry and Molecular Pharmacology, New York University School of Medicine, New York, New York, United States of America; St Vincent's Institute, AUSTRALIA

## Abstract

FANCJ/BRIP1 is an iron-sulfur (FeS) cluster-binding DNA helicase involved in DNA inter-strand cross-link (ICL) repair and G-quadruplex (G4) metabolism. Mutations in *FANCJ* are associated with Fanconi anemia and an increased risk for developing breast and ovarian cancer. Several cancer-associated mutations are located in the FeS domain of *FANCJ*, but how they affect FeS cluster binding and/or FANCJ activity has remained mostly unclear. Here we show that the FeS cluster is indispensable for FANCJ’s ability to unwind DNA substrates *in vitro* and to provide cellular resistance to agents that induce ICLs. Moreover, we find that FANCJ requires an intact FeS cluster for its ability to unfold G4 structures on the DNA template in a primer extension assay with the lagging-strand DNA polymerase delta. Surprisingly, however, FANCJ variants that are unable to bind an FeS cluster and to unwind DNA *in vitro* can partially suppress the formation of replisome-associated G4 structures that we observe in a *FANCJ* knock-out cell line. This may suggest a partially retained cellular activity of FANCJ variants with alterations in the FeS domain. On the other hand, *FANCJ* knock-out cells expressing FeS cluster-deficient variants display a similar–enhanced–sensitivity towards pyridostatin (PDS) and CX-5461, two agents that stabilise G4 structures, as *FANCJ* knock-out cells. Mutations in *FANCJ* that abolish FeS cluster binding may hence be predictive of an increased cellular sensitivity towards G4-stabilising agents.

## Author summary

Breast and ovarian cancers are often linked to a genetic predisposition, most commonly through mutations in the breast cancer susceptibility genes *BRCA1* and *BRCA2*, but also other genes, such as *FANCJ/BRIP1*, are associated with an increased disease risk. The small molecule CX-5461 is currently in phase I/II clinical trials for patients with BRCA-deficient tumours. It was originally identified as an rDNA transcription inhibitor, but–more recently–also found to bind and stabilise G4 DNA secondary structures. In this study we show that FANCJ-deficient cells have an increased sensitivity towards CX-5461. Our data further suggest that single amino acid changes in FANCJ that abolish its helicase activity or its ability to bind an iron-sulfur co-factor are sufficient to render cells more sensitive to CX-5461 treatment. Combined, our findings support a model whereby FANCJ resolves G4 structures in the context of the replisome to allow replication through guanine-rich regions of the genome. Mechanistically, FANCJ’s ability to resolve G4 structures largely depends on an intact helicase domain and partially on the iron-sulfur cluster-binding domain. The latter finding is important since a number of cancer-associated mutations are located within the iron-sulfur domain of *FANCJ*.

## Introduction

Fanconi anemia group J protein (FANCJ) was initially identified as an interaction partner of breast cancer type 1 susceptibility protein (BRCA1) and therefore termed BRCA1-interacting protein 1 (BRIP1) or BRCA1-associated C-terminal helicase 1 (BACH1) [1]. Later on, biallelic mutations in *FANCJ* were found to be cause of disease in a subset of Fanconi anemia (FA) patients [2–5]. Fanconi anemia is a chromosome instability and cancer-prone disorder that has so far been linked to mutations in 21 genes [6]. FA proteins work together in the so-called FA pathway that promotes ICL repair by the interplay of lesion excision, translesion synthesis and homologous recombination (HR) [7]. FANCJ was shown to be part of the downstream factors [2], but–somewhat surprisingly–its function in the FA pathway seems to be independent of the interaction with the HR factor BRCA1, whereas it depends on the interaction with the mismatch repair protein MLH1 and a functional helicase domain [8].

FANCJ has also been reported to have FA pathway-independent functions and has in particular been implicated in the resolution of secondary structures, most notably G4 structures [9–14]. *In vitro* FANCJ is able to unwind G4 structures [12,14], and a variety of other DNA substrates [15], in an ATP-dependent manner with a 5´-3´polarity.

**Fig 1:**
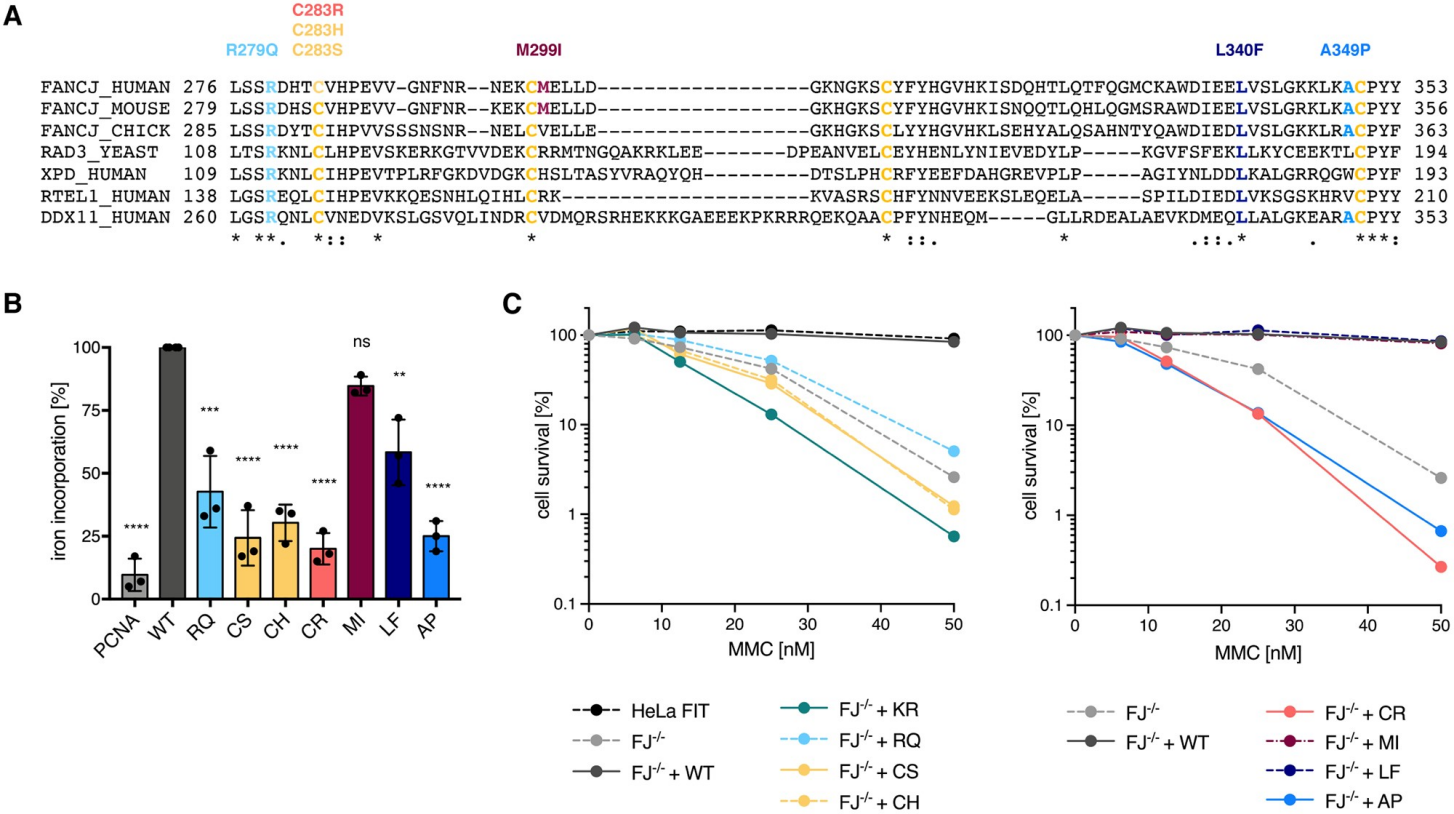
FANCJ coordinates an FeS cluster that is essential for MMC resistance. (A) Alignment of FANCJ sequences from a variety of species. FeS cluster-coordinating cysteines are depicted in yellow, disease-associated and rationally designed alterations are highlighted in colour and annotated. (B) Radioactive iron-55 incorporation by FANCJ variants, as measured by liquid scintillation counting. Levels are expressed as % iron incorporation, with wild-type levels set to 100%. The non-FeS protein PCNA was used as a negative control. Error bars depict standard deviations from three independent experiments. Statistical analysis: ordinary one-way ANOVA (****, *p* < 0.0001; ***, *p* < 0.001; **, *p* < 0.01; ns, non-significant). (C) MMC sensitivity of *FANCJ* HeLa FIT knock-out cells (FJ^–/–^) complemented with different *FANCJ* constructs. Graph depicts mean values of three independent experiments. For raw values, standard deviations and statistical analysis see S1 Table. WT, wild-type; KR, K52R; RQ, R279Q; CS, C283S; CH, C283H; CR, C283R; MI, M299I; LF, L340F; AP, A349P. See also S1 Fig.

FANCJ belongs to the Rad3 family of SF2 helicases and shares with the other members a conserved motif in the helicase domain that encompasses four cysteine residues that can coordinate a [4Fe-4S] cluster (Fig 1A) [16]. In the related helicase XPD/Rad3 the FeS cluster has been shown to act as a structural, wedge-like, element required for DNA unwinding [16–19]. A similar function has also been suggested for the FeS cluster in FANCJ, based on biochemical data with the FA-associated variant FANCJ A349P, in which replacement of a moderately conserved alanine next to one of the FeS cluster-binding cysteines leads to reduced protein-associated iron levels [4,16,20]. A more detailed characterisation of the FeS cluster in FANCJ is however missing.

Interestingly, a number of mutations in the FeS domain (Fig 1A) have been associated with cancer predisposition [21–23]. c.897G>A was found in a patient with early-onset breast cancer and gives rise to FANCJ M299I, a hyper-active variant with increased helicase activity [1,21,24]. Later on, a screen for *FANCJ* mutations in a Korean cohort with *BRCA1/2* mutation-negative high-risk breast cancer identified the germline mutation c.1018C>T, giving rise to FANCJ L340F, as likely pathogenic [22]. More recently, targeted sequencing of 94 cancer-predisposing genes in a patient cohort with early-onset/ familial prostate cancer led to the discovery of a germline mutation (c.847T>C) giving rise to FANCJ C283R that was classified as potentially pathogenic [23]. So far, however, it has remained mostly unclear how these mutations affect FeS cluster binding and FANCJ function.

Here we show that some, but not all, cancer-associated mutations in the FeS domain of *FANCJ*, affect FeS cluster coordination and, presumably as a consequence thereof, DNA unwinding and sensitivity to the ICL-inducing agent mitomycin C (MMC) and the G4-stabilising agents PDS and CX-5461. We further show that FANCJ can unwind parallel G4 structures *in vitro* and allow DNA replication past these structures by DNA polymerase delta (Pol δ). Accordingly, FANCJ prevents the formation of replisome-associated G4 structures *in vivo*. Surprisingly, however, while in our experimental conditions G4 resolution *in vitro* strictly requires an FeS cluster, FANCJ’s ability to prevent the accumulation of G4 structures at replisomes only partially depends on the FeS domain, suggesting that FANCJ may retain some of its activities in the absence of an intact FeS domain.

## Results

### FeS cluster binding is required for FANCJ’s role in ICL repair

To better understand the function of the FeS cluster in FANCJ, we prepared constructs for a number of FANCJ variants (Fig 1A). In the rationally designed variants FANCJ C283S and C283H, the first FeS cluster-coordinating cysteine was changed to serine and histidine, respectively, which should abolish or reduce FeS cluster binding. FANCJ R279Q was included because the homologous arginine residues in the related helicases DDX11 and XPD are required for FeS cluster stabilisation, and mutations causing their alteration are linked to Warsaw Breakage Syndrome and Trichothiodystrophy, respectively [16,25–27]. Moreover, the FA-associated variant A349P, known to display reduced protein-associated iron levels [4,16,20], was added as a variant that is most likely FeS cluster-deficient.

To investigate whether cancer-associated mutations in the FeS domain affect FeS cluster binding, we included FANCJ C283R, associated with early-onset prostate cancer and classified as potentially pathogenic [23]; FANCJ L340F, associated with familial breast cancer and predicted to be likely pathogenic [22]; and the previously studied FANCJ M299I, found in a patient with early-onset breast cancer and proposed to be hyper-active [1,21,24].

In an iron incorporation assay using radioactive iron-55 (Fig 1B; S1 Fig), FANCJ C283S and C283H displayed greatly reduced iron incorporation as compared to wild-type FANCJ, demonstrating that cysteine-283 is essential for FeS cluster binding and that a histidine residue at this position cannot substitute for cysteine. In agreement with a previous study that reported a reduced iron content [20], FANCJ A349P displayed a similar reduction in iron incorporation as the cysteine variants, suggesting that it is devoid of an FeS cluster. Iron incorporation was also severely reduced in FANCJ R279Q, albeit not to the same extent as in the variants above, which may be suggestive of unstable FeS cluster binding. Interestingly, iron incorporation was affected to different degrees in the three cancer-associated variants. While FANCJ M299I displayed close-to wild-type levels of iron incorporation and FANCJ L340F retained an intermediate level of iron incorporation, FANCJ C283R was largely unable to bind an FeS cluster.

We then decided to study side-by-side the ability of the different variants to complement a FANCJ-deficient cell line. To this end, using CRISPR/Cas9 we generated a *FANCJ* HeLa Flp-In T-REx (HeLa FIT) knock-out cell line that can be complemented with different *FANCJ* variants in a doxycycline-dependent manner. It is of note that the levels of the exogenously expressed *FANCJ* constructs are slightly lower than the level of endogenous FANCJ (S1 Fig).

Using this deletion/complementation system we studied sensitivity to the ICL-inducing agent mitomycin C (MMC). As reported previously [2,3], FANCJ-deficient cells were highly sensitive to MMC (Fig 1C). Importantly, cells complemented with wild-type *FANCJ* showed the same resistance to MMC as the parental HeLa FIT cell line, establishing the utility of our deletion/complementation system for probing FANCJ variants. Strikingly, MMC sensitivity correlated largely with FeS cluster-binding status in that all *FANCJ* knock-out cell lines expressing FeS cluster-deficient variants were as sensitive, or even more sensitive, to MMC as the *FANCJ* knock-out cell line itself (Fig 1C). Moreover, complementation with the R279Q variant rendered *FANCJ* knock-out cells slightly less sensitive to MMC, which is in agreement with the reduced, but not completely abolished, *in vitro* iron incorporation of this variant (Fig 1B). Notably, *FANCJ* knock-out cells complemented with the cancer-associated variant FANCJ C283R were highly sensitive to MMC, similarly to the knock-out cell line expressing the helicase-dead variant FANCJ K52R [1]. In contrast, expression of FANCJ L340F, which had a partial defect in FeS cluster binding *in vitro*, or the FeS cluster-containing M299I variant restored MMC sensitivity of *FANCJ* knock-out cells to the same extent as expression of the wild-type construct (Fig 1C).

Taken together, our findings establish that FeS cluster binding in FANCJ can be influenced by non-coordinating residues in the FeS domain and that FeS cluster binding status largely correlates with MMC sensitivity, suggesting that the FeS cluster in FANCJ is required for ICL repair and/or signalling.

### FeS cluster binding is important for DNA unwinding

**Fig 2:**
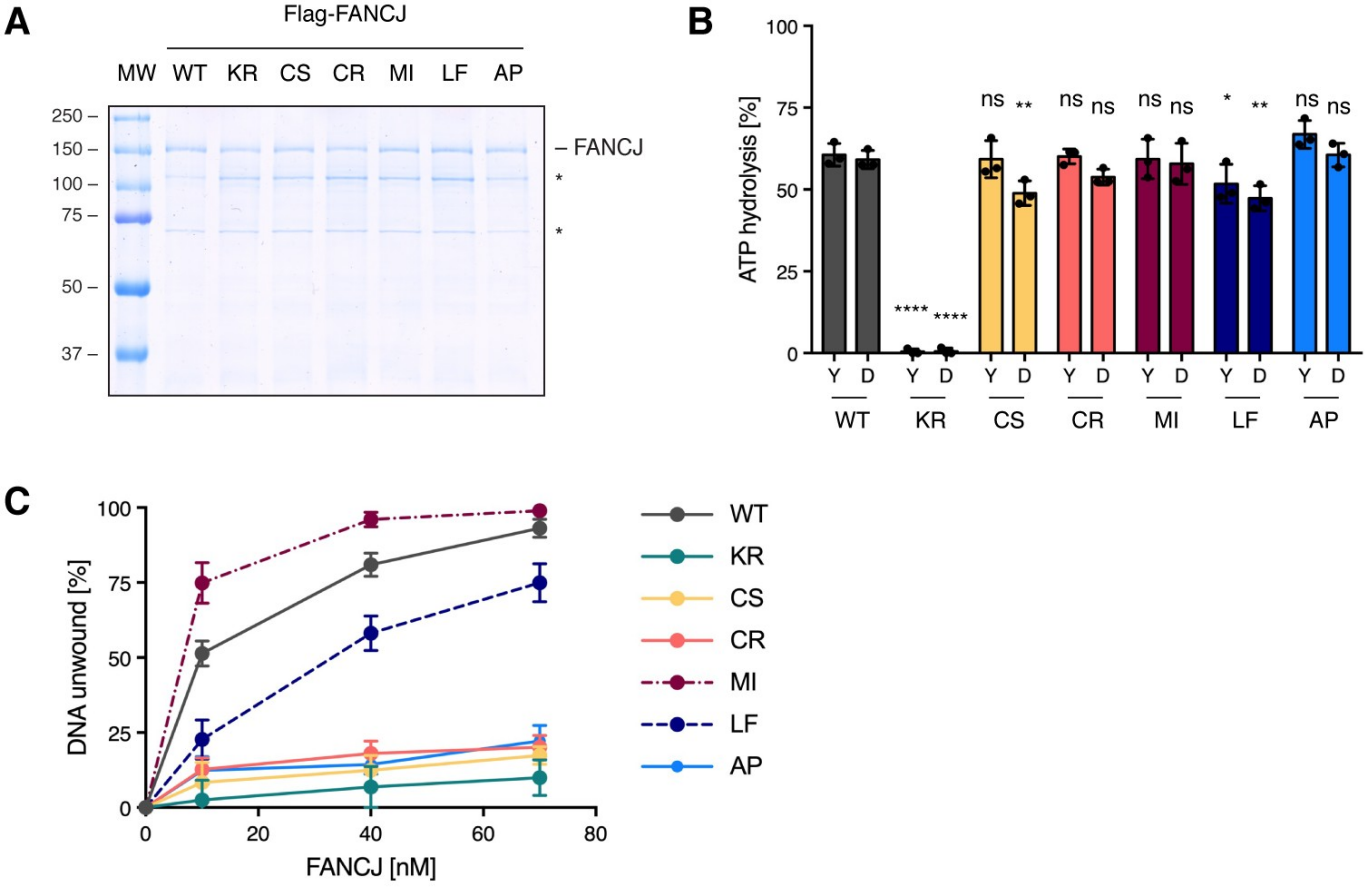
The FeS cluster in FANCJ is indispensable for helicase activity. (A) InstantBlue stained SDS-PAGE gel of purified N-terminally Flag-tagged FANCJ variants. Asterisks mark contaminants. (B) ATP hydrolysis of FANCJ variants in the presence of an oligonucleotide-based Y-structure (Y) or D-loop (D) substrate, as measured by the release of inorganic phosphate from radio-labelled γ-^32^P-ATP in thin-layer chromatography. Activity is depicted as % of hydrolysed ATP, with background activity in the absence of DNA subtracted. Error bars depict standard deviations from three independent experiments. Statistical analysis: ordinary one-way ANOVA (****, *p* < 0.0001; **, *p* < 0.01; *, *p* < 0.1; ns, non-significant). (C) Graphical representation of DNA unwinding of a D-loop substrate with increasing concentrations of FANCJ variants. In the graph, mean values and standard deviations of three independent experiments are depicted. WT, wild-type; KR, K52R; CS, C283S; CR, C283R; MI, M299I; LF, L340F; AP, A349P. See also S2 Fig.

To determine how the biochemical activities of the cancer-associated FANCJ variants are influenced by the FeS cluster-binding status, we purified the different variants as N-terminally Flag-tagged proteins from *Sf9* insect cells (Fig 2A). In addition to the disease-associated variants and the wild-type protein, we included the FeS cluster-deficient FANCJ C283S and the helicase-dead variant FANCJ K52R. We then tested ATPase activity and DNA binding. With the exception of FANCJ K52R, all FANCJ variants were able to hydrolyse ATP to a similar extent as the wild-type protein, both in the presence of oligo-based Y-structure DNA and a D-loop substrate (Fig 2B; S2 Fig). All variants, including FANCJ K52R, were also able to bind a Y-structure DNA substrate (S2 Fig), suggesting that neither ATP hydrolysis nor an intact FeS domain is required for DNA binding.

In contrast, the FANCJ variants C283S, C283R and A349P, with diminished iron levels (Fig 1B), exhibited defective unwinding of a D-loop-like DNA substrate, which was similar to the helicase-dead control (Fig 2C; S2 Fig). Moreover, correlating with its reduced ability to incorporate iron (Fig 1B), FANCJ L340F displayed a reduced DNA unwinding activity (Fig 2C; S2 Fig). As for the FeS cluster-containing M299I variant, we observed wild-type levels of DNA binding and ATP hydrolysis (Fig 2B; S2 Fig), whereas DNA unwinding was increased as compared to the wild-type protein (Fig 2C; S2 Fig). While these results differ from previous studies that report not only an increased helicase activity, but also an increase in ATP hydrolysis [21,28], these differences likely arise from different protein production and purification protocols.

Taken together, we show that FANCJ’s ability to unwind DNA correlates perfectly with its FeS cluster-binding status, whereas DNA binding and ATP hydrolysis are largely unaffected by the presence or absence of an FeS cluster. These results could explain why we observe a stronger MMC sensitivity in *FANCJ* knock-out cells expressing helicase- or FeS cluster-deficient variants of *FANCJ*, than in *FANCJ* knock-out cells (Fig 1C). Given that these variants can bind DNA without being able to unwind it, they may form non-productive complexes with DNA and, hence, block the access of alternative DNA processing factors.

These findings are also in line with what has been reported for the related helicase XPD where the FeS cluster was shown dispensable for DNA binding and ATP hydrolysis but required for helicase activity [16–19]. Mechanistically, the FeS cluster in XPD is thought to act as a wedge-like element to unwind DNA [17–19]. Interestingly, however, loss of the FeS cluster in another family member, DDX11, does not only abolish DNA unwinding, but also DNA binding and ATPase activity [27]. Hence, even in closely related proteins the function of the FeS cluster can vary.

### FeS cluster binding is required for FANCJ’s ability to resolve G4 structures ahead of Pol δ

**Fig 3:**
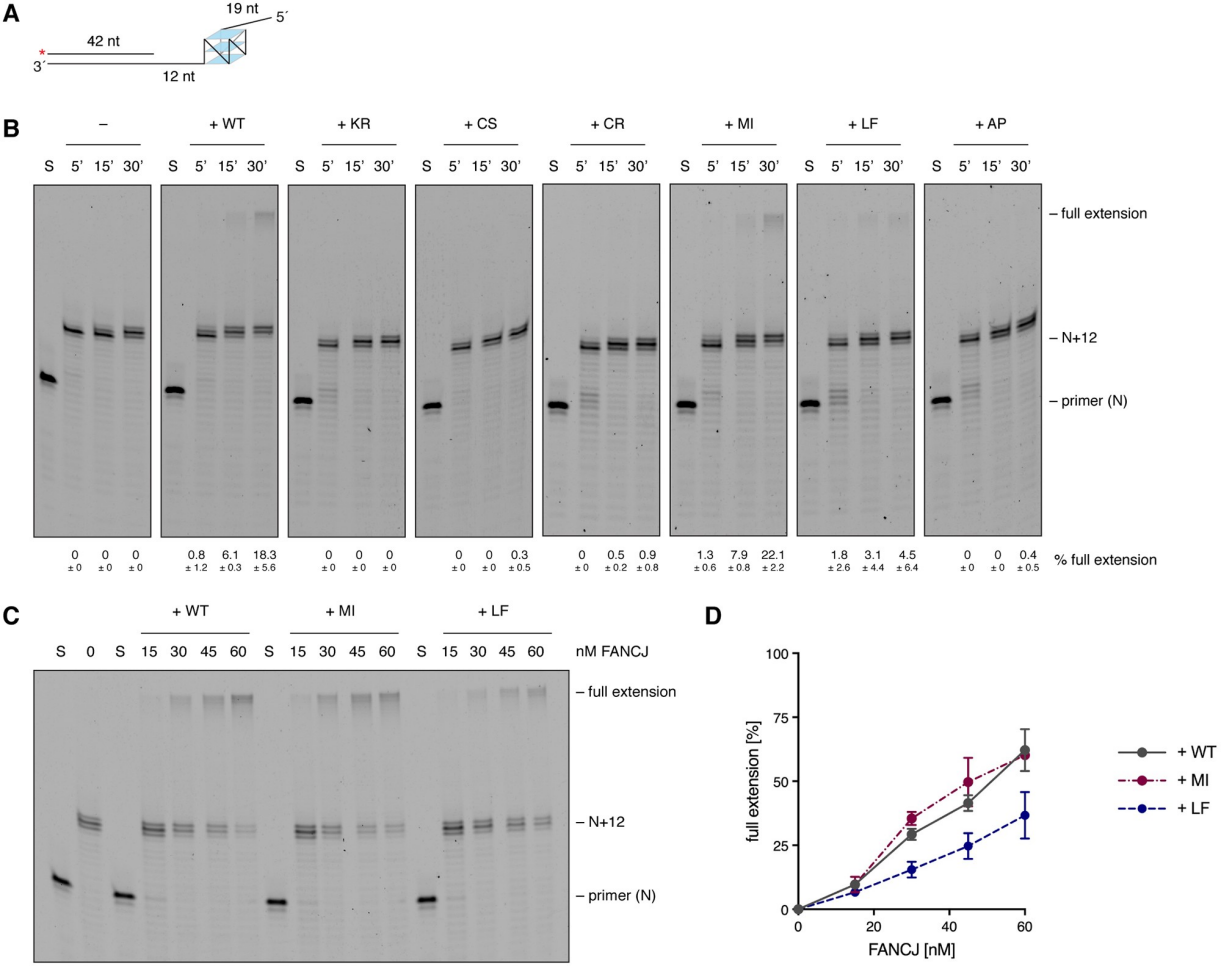
FANCJ can resolve G4 structures ahead of Pol δ *in vitro*. (A) Scheme of primer-template substrate with a sequence forming a parallel G4 structure on the template strand ahead of the primer that can be extended by Pol δ. Asterisk indicates 5´-FAM label. Numbers indicate lengths of primers and gaps in nucleotides (nt). (B) Time-resolved primer extension assay with 10 nM of Pol δ alone (–) and in the presence of 35 nM FANCJ wild-type or variants. Numbers indicate the mean percentage of full extension and standard deviations from two independent experiments. (C,D) Primer extension assay with 10 nM of Pol δ and increasing amounts of different FANCJ variants (C) and quantification (D). In the graph, mean values and standard deviations of two independent experiments are depicted. N+12 denotes position of G4 block relative to 3´-end of primer. S, DNA substrate without protein; WT, wild-type; KR, K52R; CS, C283S; CR, C283R; MI, M299I; LF, L340F; AP, A349P.

We next sought to determine the requirement of the FeS cluster for FANCJ’s ability to unwind complex, and physiologically relevant, DNA secondary structures, such as G-quadruplexes. To this end, we designed a DNA primer-template substrate with a parallel G4 structure located ahead of the primer-template junction on the templating strand (Fig 3A).

When the lagging-strand DNA polymerase Pol δ was incubated with this substrate, it was able to extend the primer by approximately 12 nucleotides until the beginning of the G4 structure-forming region that constituted a complete roadblock (Fig 3B). This roadblock was alleviated in the presence of FANCJ, where Pol δ was able to extend the primer past the G4 structure and to achieve full extension (Fig 3B). Importantly, G4 bypass in our experimental conditions was strictly dependent on helicase activity and an intact FeS domain since neither the helicase-dead variant K52R nor the FeS cluster-deficient variants C283S, C283R and A349P, were able to promote primer extension by Pol δ past the G4-forming region (Fig 3B).

To better compare wild-type FANCJ with the active or partially active M299I and L340F variants (Fig 3B), we then performed concentration-dependent primer extension assays (Fig 3C and 3D). In this setup, FANCJ L340F was able to promote full primer extension, although to a lesser extent than the wild-type protein, which is in line with its reduced DNA unwinding activity (Fig 2C; S2 Fig). Despite its increased activity observed in D-loop unwinding (Fig 2C; S2 Fig), FANCJ M299I was however not more proficient in allowing G4 bypass than the wild-type protein.

### FANCJ’s FeS cluster plays a role in cellular G4 metabolism

**Fig 4:**
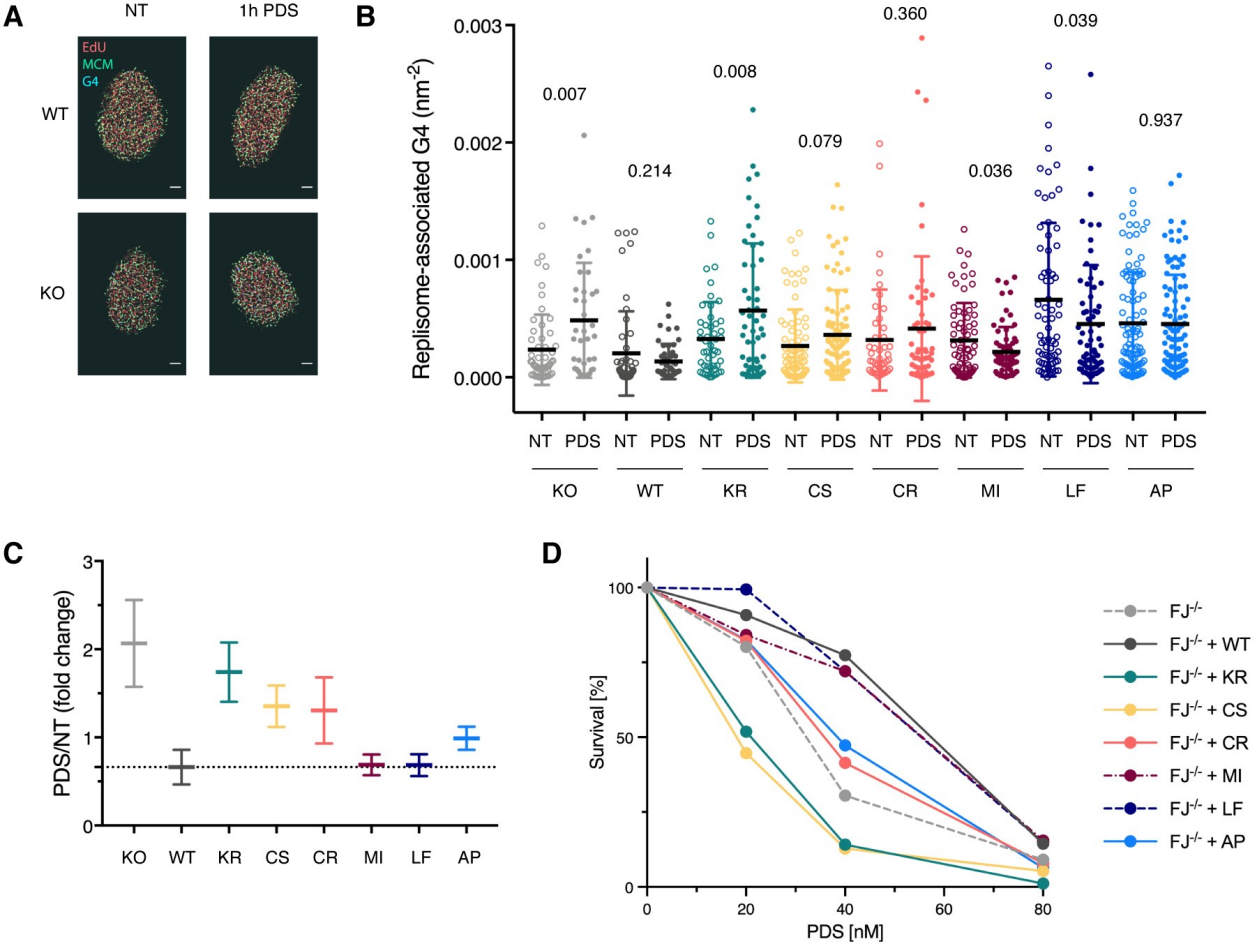
The FeS cluster is required to efficiently prevent replisome-associated G4 structures. (A) Representative SMLM images labelled for nascent DNA (red), G4 structures (blue), and MCM (green) to detect replisome-associated G4 structures. Scale bar, 2 μm. (B) Densities of replisome-associated G4 structures in *FANCJ* knock-out cells (KO) complemented with the indicated variants. Each cell line was either non-treated (NT) or treated for 1h with 20 μM PDS. Individual data point represents result from one nucleus. Black horizontal line and error bars indicate mean ± standard deviation. Values on graph indicate *p*-values of unpaired two-sample t-tests between NT and PDS-treated cells. Note that for technical reasons not all cell lines were treated in parallel, and, hence, absolute values between cell lines were not compared. For raw values and analysis see S2 Table. (C) Averaged fold change of the densities of replisome-associated G4 structures in PDS-treated compared to non-treated cells. Bold horizontal lines represent the ratio between the mean values of PDS-treated and NT cells from (B), and the error bars indicate the propagated standard errors of the mean. (D) Sensitivity of *FANCJ* HeLa FIT knock-out cells (FJ^–/–^) complemented with different *FANCJ* constructs to PDS treatment. In the graph, the mean values of three independent experiments are depicted. For raw values, standard deviations and statistical analysis see S3 Table. WT, wild-type; KR, K52R; CS, C283S; CR, C283R; MI, M299I; LF, L340F; AP, A349P.

Our data so far demonstrate that proper coordination of FANCJ’s FeS cluster is required for its ability to unwind G4 structures ahead of Pol δ *in vitro*. To address whether FANCJ’s G4 resolution activity is also relevant during cellular DNA replication, we made use of multi-colour single-molecule localisation microscopy (SMLM) combined with data-mining algorithms [29] to enable quantification of the association of G4 structures with replisomes. To this end, we used a triple-labelling strategy to detect the replicative helicase MCM, DNA G4 structures, and the sites of nascent DNA synthesis by EdU staining (Fig 4A).

To study the impact of FANCJ on stabilised G4 structures at DNA replication forks, we treated cells for 1h with the G4-stabilising agent PDS [30] and analysed the ratio of replisome-associated G4 structures in PDS-treated over non-treated cells (Fig 4B and 4C). In line with a role of FANCJ in G4 metabolism, when treated with PDS, *FANCJ* knock-out cells displayed a significant increase in the amount of replisome-associated G4 structures, which was suppressed by the expression of wild-type *FANCJ* and by a construct giving rise to the catalytically active variant FANCJ M299I (Fig 4B and 4C). Interestingly, FANCJ L340F was also able to fully suppress the accumulation of replisome-associated G4 structures (Fig 4B and 4C), although *in vitro* it appeared less active than wild-type FANCJ and the M299I variant (Fig 3B–3D). This is–on the other hand–perhaps not so surprising when taking into consideration the varied stability of cellular G4 structures [31–33] as compared to the highly stable structure used in our *in vitro* assay.

It is of note that in cells complemented with wild-type FANCJ or the M299I and L340F variants, the amount of replisome-associated G4 structures upon PDS treatment is decreased as compared to non-treated cells. While this may seem counter-intuitive, we speculate that background G4 levels, as found in non-treated samples, are tolerated by the cell, whereas G4 levels that reach a certain threshold may elicit a checkpoint response that reduces the amount of newly activated replisomes and leads to a preferential removal of G4 structures in active replisomes.

In contrast to wild-type FANCJ, complementation of the knock-out cells with the helicase-dead variant K52R did not, or only marginally, reduce the amount of replisome-associated G4 structures upon PDS treatment, suggesting that FANCJ’s role in G4 resolution is largely dependent on its helicase activity. To our surprise, however, complementation of the knock-out cells with the FANCJ C283S, C283R and A349P variants could partially suppress the formation of replisome-associated G4 structures (Fig 4B and 4C), although in our *in vitro* experiments these variants lacked any detectable helicase activity (Fig 2C) and were deficient in providing G4 bypass (Fig 3B). This may suggest that variants that are unable to coordinate an FeS cluster *in vitro* could still be able to–loosely–bind an FeS cluster in a cellular context and, hence, retain some helicase activity. In favour of this idea we note that of the three variants that were FeS cluster-deficient *in vitro*, FANCJ A349P had the mildest cellular phenotype (Fig 4B and 4C), which seems intuitive given that this variant retains all four cluster-coordinating cysteines.

We next tested cellular sensitivity to PDS, and observed that *FANCJ* knock-out cells were more sensitive towards PDS than knock-out cells complemented with wild-type FANCJ (Fig 4D). In agreement with our observation that FANCJ M299I and L340F were able to fully suppress the accumulation of replisome-associated G4 structures (Fig 4B and 4C), both variants were able to complement FANCJ-deficient cells to a similar extent as the wild-type construct (Fig 4D). In contrast, *FANCJ* knock-out cells complemented with FANCJ C283R, C283S and A349P were as sensitive or even more sensitive to PDS as the knock-out cell line, which is surprising given that these variants were able to partially suppress the accumulation of replisome-associated G4 structures (Fig 4B and 4C). This may suggest that in addition to G4 resolution at replisomes, FANCJ has other functions in G4 metabolism that contribute to cell survival.

### A functional FeS domain contributes to cellular resistance to the G4-stabilising agent CX-5461

In recent years, G4 structures have been proposed as potential therapeutic targets in DNA repair-deficient cancers [34–36], which has spurred the search for G4-targeting small molecules that could be used as medicinal compounds. Interestingly, the rDNA transcription inhibitor CX-5461 [37] was recently found to bind and stabilise G4 structures, and to be cytotoxic for BRCA-deficient cancer cells [38]. CX-5461 is currently in phase I/II clinical trials for patients with BRCA-deficient tumours (NCT02719977).

**Fig 5:**
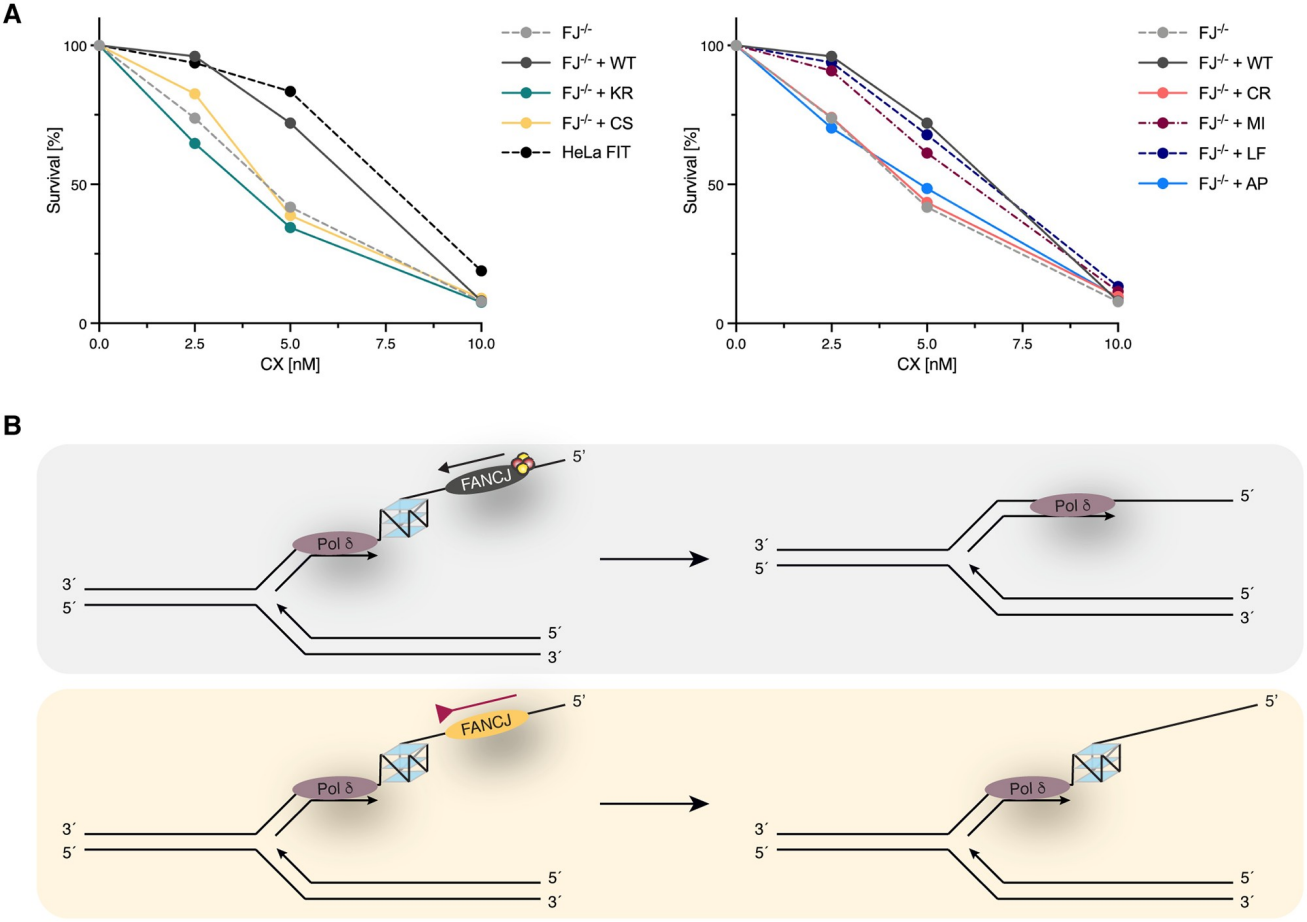
*FANCJ* knock-out cells display enhanced sensitivity to the G4-stabilising agent CX-5461. (A) Sensitivity of *FANCJ* HeLa FIT knock-out cells (FJ^–/–^) complemented with different *FANCJ* constructs to CX-5461 treatment. In the graphs, the mean values of three independent experiments are depicted. For raw values, standard deviations and statistical analysis see S4 Table. CX, CX-5461; WT, wild-type; KR, K52R; CS, C283S; CR, C283R; MI, M299I; LF, L340F; AP, A349P. (B) Model showing wild-type FANCJ able to resolve G4 structures on the templating strand during DNA replication, which would allow continuous DNA synthesis (upper panel). In contrast, the inability of FeS cluster-deficient FANCJ to efficiently resolve G4 structures would lead to polymerase stalling and persistent replisome-associated G4 structures (lower panel).

To determine whether FANCJ-deficient cells or cells with mutations in the FeS domain of *FANCJ* could also be targeted by CX-5461 treatment, we tested the sensitivity of our different cell lines towards this compound. Like PDS, CX-5461 turned out to have a narrow range of use since parental HeLa FIT cells or *FANCJ* knock-out cells complemented with wild-type *FANCJ* were 100% viable in response to a treatment with 2.5 nM CX-5461, while a treatment with 10 nM was lethal (Fig 5A). Nevertheless, we could observe a reproducibly increased sensitivity of *FANCJ* knock-out cells, and knock-out cells complemented with the helicase-dead or FeS cluster-deficient *FANCJ* variants, when compared to wild-type cells (Fig 5A). In contrast, constructs coding for both FANCJ M299I and L340F were able to fully complement the *FANCJ* knock-out cell line (Fig 5A). The latter result suggests that the slight changes in DNA unwinding that we observe *in vitro* do not translate in an increased sensitivity to CX-5461 treatment, similarly to what we observe in response to PDS (Fig 4D) and MMC (Fig 1C).

Taken together, these data indicate that FANCJ-deficient cells or cells that express variants that are helicase-dead or FeS cluster-deficient are–in addition to PDS–also sensitive to a the G4-stabilising agent CX-5461.

## Discussion

The study here presents important mechanistic insight into FANCJ’s role in G4 metabolism. Our *in vitro* experiments show that FANCJ’s helicase activity and its ability to unwind G4 structures ahead of Pol δ, depend on a functional FeS cluster. Somewhat surprisingly, however, the disease-associated variants C283R [22] and A349P [4,16,20], and the rationally designed FANCJ C283S, which are unable to coordinate an FeS cluster *in vitro*, can partially suppress the increased association of G4 structures with replisomes that we observe in *FANCJ* knock-out cells. Since the helicase-dead variant FANCJ K52R cannot, or only to a minor extent, complement *FANCJ* knock-out cells in this experiment, the ability of FANCJ to suppress the accumulation of replisome-associated G4 structures, seems to depend–to a large part–on FANCJ’s helicase activity. The most likely explanation why the FeS domain variants can partially complement *FANCJ* knock-out cells is therefore a partially preserved helicase activity *in vivo*. It is conceivable that FANCJ C283R, C283S and A349P are unable to coordinate an FeS cluster under our experimental conditions *in vitro*, while they are able to–presumably loosely–bind an FeS cluster *in vivo* and to retain some helicase activity. In line with this hypothesis, the A349P variant that retains all four cluster-coordinating cysteines and therefore seems most likely to be able to bind an FeS cluster, can suppress the accumulation of replisome-associated G4 structures to a greater extent than the C283 variants. It should also be noted that cellular G4 structures are of varied stability [31–33], whereas the parallel G4 structure used in our *in vitro* assays is highly stable, which may have precluded the detection of any remaining G4-unwinding activity.

On the other hand, *FANCJ* knock-out cells complemented with the C283S, C283R and A349P variants are as sensitive to the G4-stabilising agents PDS and CX-5461 as the knock-out cell line. This may suggest that in addition to G4 resolution at replisomes, FANCJ has other functions in G4 metabolism that contribute to cell survival, *e*.*g*. in checkpoint activation, as reported previously in response to hydroxyurea [39]. Interestingly, several recent studies have demonstrated that the cytotoxic effects of PDS and CX-5461 involve topoisomerase II poisoning [40–42]. It may therefore also be possible that the reduced helicase activity of the C283S, C283R and A349P variants allows for a partial suppression of G4 accumulation at replisomes, but is insufficient to preclude the cytotoxic effects of topoisomerase II trapping through the remaining G4 structures.

The situation is different for FANCJ L340F [22], in which the highly conserved lysine-340 is replaced by a bulky phenylalanine residue. While this variant displays reduced iron incorporation and a reduced ability to unwind DNA or provide G4 bypass *in vitro*, in a cellular context, it can suppress the accumulation of PDS-induced replisome-associated G4 structures and fully complement *FANCJ* knock-out cells with respect to MMC, PDS and CX-5461 sensitivity. Also the third cancer-associated variant studied here, FANCJ M299I, associated with early-onset breast cancer [1], was able to complement *FANCJ* knock-out cell lines in all experiments to a similar extent as the wild-type construct, despite an altered–in this case increased–unwinding activity. It would therefore appear that the slight changes in helicase activity observed with the M299I and L340F variants *in vitro* do not affect FANCJ’s function in a cellular context.

Combined, our study establishes an important role for FANCJ and its FeS cluster in the resolution of G4 structures *in vitro* and *in vivo* (Fig 5B). Moreover, we provide evidence that cells depend on FANCJ and FeS cluster coordination for enhanced resistance to the G4-stabilising agents PDS and CX-5461, the latter being currently in phase I/II clinical trials. This may suggest that patients with FANCJ-deficient tumours could possibly be considered for CX-5461 treatment in the future.

## Material and methods

### Plasmid DNA and site-directed mutagenesis

*FANCJ* cDNA was purchased from GE Healthcare Dharmacon and used as a PCR template. All plasmids used in this study are based on the GATEWAY system (Invitrogen). GATEWAY destination vectors allowed expression of N-terminally Flag-tagged constructs in human and insect cells under control of a cyto-megalovirus (CMV) or polyhedrin promoter respectively.

In order to introduce nucleotide changes in the entry clone *FANCJ*-pDONR221, primers containing the desired mutations were designed according to the guidelines of the QuikChange Site-Directed Mutagenesis manual (Stratagene). Successful site-directed mutagenesis was verified by sequencing (Microsynth).

### *Sf9* insect cells and baculoviruses

*Spodoptera frugiperda* (*Sf9*) cells were cultured in HyClone SFX-insect medium (GE Healthcare), usually as liquid cultures on a horizontal shaker at 120 rpm and 27°C. The Bac-to-Bac Baculovirus Expression System (Invitrogen) was used to generate bacmids and baculoviruses.

### Recombinant protein expression and purification

For protein expression and purification, a 500 ml culture of *Sf9* cells at 2x10^6^ cells/ml was infected at an estimated multiplicity of infection (MOI) of 1 with recombinant baculoviruses encoding for N-terminally Flag-tagged FANCJ WT or FANCJ variants. 48h after infection, the cells were spun at 475 x g for 20 minutes at 4°C. The pellet was lysed for 1h in 3 packed-cell volumes (PCV) of buffer A (50 mM Na_2_HPO_4_/NaH_2_PO_4_ (pH 7.4), 150 mM NaCl, 10% glycerol, 0.01% NP-40, 0.5 mM EDTA, 1% Triton X-100) supplemented with protease inhibitors (Roche). Lysed cells were spun for 30 minutes at 17’000 x g and 4°C. The supernatant was incubated on Flag M2 beads (Sigma-Aldrich) for 2h at 4°C. Subsequently, the beads were washed twice with buffer B (50 mM Na_2_HPO_4_/NaH_2_PO_4_ (pH 7.4), 150 mM NaCl, 10% glycerol, 0.01% NP-40, 0.5 mM EDTA), followed by one wash with buffer A, and one wash with buffer C (50 mM Na_2_HPO_4_/NaH_2_PO_4_ (pH 7.4), 150 mM NaCl, 10% glycerol, 0.01% NP-40, 0.5 mM EDTA, 5 mM MgCl_2_,) supplemented with 5 mM ATP. Finally, the beads were washed extensively in buffer B and eluted for 1h in buffer B containing 200 μg/ml 3x Flag peptide (Sigma-Aldrich). Purified proteins were filtered through Micro Bio-Spin columns (BioRad), aliquoted, snap-frozen and stored at -80°C.

To estimate purity and quantity, proteins were separated on a 10% SDS PAGE and stained with InstantBlue (Expedeon). Protein concentrations were calculated using a BSA standard curve.

Human polymerase δ was co-expressed with PCNA in *Sf9* insect cells, and purified *via* a Flag-tag on POLD1, as described previously [43].

### Iron incorporation assay

For iron incorporation assays, 30 ml of *Sf9* cells (at 2x10^6^ cells/ml) were infected with FANCJ baculoviruses in normal growth medium supplemented with 0.7 mM NaAscorbate and 0.7 μCi/mL of ^55^FeCl_3_ (Perkin Elmer) for 48h.

The cells were collected by centrifugation at 475 x g and 4°C for 5 min, washed with citrate buffer (50 mM Citrate and 1 mM EDTA in 1x PBS, pH 7.0) followed by a PBS wash. The cells were then lysed with 3 PCV of buffer A for 30 minutes at 4°C and spun for 30 minutes at 17’000 x g and 4°C. The supernatant was immunoprecipitated on Flag-M2 beads (Sigma-Aldrich) for 2h. Subsequently, the beads were washed in buffer B and A followed by a buffer C wash. Lastly, the beads were returned to buffer B and eluted in buffer B supplemented with 200 μg/ml 3x Flag peptide (Sigma-Aldrich).

80% of the elution was mixed with 1 ml of Ultima Gold scintillation liquid (Perkin Elmer). Counts per minute (cpm) were measured with a Tri-Carb scintillation counter (Packard) using standard ^3^H settings. The remaining elution was run on a gradient SDS-PAGE (Roche), and proteins were stained with InstantBlue (Expedeon) and quantified using the ImageJ software [44]. Sample counts were then normalised to the protein amount.

### DNA substrates

DNA oligonucleotides were purchased from Microsynth. Sequences and modifications are listed in S5 Table. For the generation of Y-structure and D-loop substrates, 200 nM of the FAM-labelled oligonucleotide were incubated with 300 nM of the unlabelled oligonucleotide(s) in 10 mM Tris-HCl (pH 8.0), 50 mM NaCl and 10 mM MgCl_2_. Substrates were annealed using a PCR cycler (Biometra) program. Briefly, the samples were heated to 95°C for 5 minutes following a step-wise reduction in temperature (minus 5K every 3 min) to 4°C. G4-containing substrates were annealed in 10 mM Tris-HCl (pH 8.0), 50 mM KCl and 10 mM MgCl_2_, boiled for 10 minutes at 95°C, allowed to slowly cool down to room temperature, and stored at 4°C until use.

### Electrophoretic mobility shift assay

To analyse binding of FANCJ to DNA substrates, different concentrations of FANCJ and its variants were incubated with 5 nM of a Y-structure substrate in a 10 μl reaction volume of buffer D (15 mM Na_2_HPO_4_/NaH_2_PO_4_ (pH 7.4), 45 mM NaCl, 3% glycerol, 40 mM Tris HCl (pH 7.4), 25 mM KCl, 100 ng/ul BSA, 2 mM DTT, 0.1 mM EDTA). After a 30 min-incubation at 25°C, an equal volume of 2x EMSA loading dye (7% Ficoll, 20 mM Tris-HCl pH 8.0, 20 mM EDTA) was added to the reaction. Samples were run on a non-denaturing polyacrylamide gel (1x TBE, 5% PAA, Acrylamide/ Bisacrylamide ratio 19:1) for 45 minutes at 80 V in 1x TBE buffer. Gels were scanned with a Typhoon FLA9500 laser scanner (GE Healthcare) using the fluorescence imaging mode.

### Helicase assay

To assess DNA unwinding, helicase assays were performed in a 10 μl reaction in buffer D. Different concentrations of FANCJ were pre-incubated with 2.5 nM of a D-loop substrate for 15 minutes on ice. Subsequently, the mix was supplemented with 5 mM MgCl_2_, 2 mM ATP and 50 nM competitor DNA and incubated for 30 minutes at 30°C. The reactions were stopped by adding 2x helicase loading dye (7% Ficoll, 20 mM Tris-HCl pH 8.0, 20 mM EDTA, 0.2% SDS, 2 mg/ml Proteinase K (Fermentas) and put on ice before being separated on a 7% non-denaturing PA-gel (1xTBE, 7% PAA, Acrylamide/ Bisacrylamide ratio 19:1) for 1h at 80V in 1x TBE. Gels were scanned with a Typhoon FLA9500 laser scanner (GE Healthcare) using the fluorescence-imaging mode, and quantified using the ImageJ software [44].

### ATPase assay

ATPase activity was measured by the release of inorganic phosphate from radiolabelled γ-^32^P-ATP (Perkin Elmer). Reactions were carried out in a 10 μl reaction volume and contained 50 nM DNA substrate, 5 mM MgCl_2_, 0.033 μM γ-^32^P-ATP, 0.01 mM cold ATP and 70 nM of protein. The reaction was incubated for 30 minutes at 37°C and subsequently stopped with EDTA at a final concentration of 50 mM. The released phosphate was separated from ATP by thin-layer chromatography (TLC). To this end, 1/10 of the reaction was spotted onto a TLC plate (Merck Millipore) and placed into the carrier solvent (0.15 M LiCl and 0.15 M Formic acid). The plates were air-dried, wrapped in clingfilm and the signal was transferred onto a storage phosphor screen (GE Healthcare), which was then imaged with a Typhoon FLA9500 scanner (GE Healthcare) using the autoradiography imaging mode. Quantification was done using the ImageJ software [44].

### Primer extension assay

Primer extension assays were carried out in a reaction volume of 20 μl in 10 mM Tris (pH 8.0), 0.1 mM DTT, 25 mM potassium acetate, 8 mM MgCl_2_, 0.1 mg/ml BSA, 1 mM ATP, 0.1 mM dNTP mix, 20 nM of the primer-template substrate, and the indicated amounts of FANCJ. The reaction was started by adding 10 nM of Pol δ and incubated for 30 minutes at 37°C. The primer extension reaction was stopped with an equal volume of a 2x STOP solution (10 mM EDTA in formamide, Bromophenol Blue and 400 nM of competitor DNA). Subsequently, the extensions were resolved on a denaturing PA-urea gel (1x TBE, 7 M urea, 12% PAA, Acrylamide/ Bisacrylamide ratio 19:1) in 1x TBE run at 20 W for 2h. Gels were imaged with a Typhoon FLA9500 scanner (GE Healthcare) and quantified with ImageJ [44].

### Human cells

Human cervix epithelioid carcinoma Flp-In T-REx (HeLa FIT) cells [45] were routinely cultured in Dulbecco's modified Eagle's medium (DMEM) (ThermoFisher 11965) with 5% fetal calf serum (FCS) (Gibco 10270) inside a 37°C incubator at a 5% CO_2_-containing atmosphere.

*FANCJ* was knocked out in HeLa FIT cells using the CRISPR/Cas9 system [46]. The single guide RNA (sgRNA) was designed to target Exon 2 (GTCTGAATATACAATTGGTG) using an online tool (http://crispr.mit.edu). To do so, the sgRNA was transfected together with the plasmid encoding Cas9 using Lipofectamine 2000 (Thermo Fisher Scientific) according to the manufacturer's protocol. 48h after transfection, single clones were isolated and grown. Clones were analysed by Western blot for the presence of FANCJ using an antibody targeting the N-terminal part of the protein. Promising clones were then further analysed by an endogenous IP. The cells were also tested by a Mass Spectrometry (MS) approach for the absence of FANCJ.

Verified *FANCJ* knock-out (KO) clones were transfected with plasmids encoding for FANCJ WT and variants in the presence of an Flp-In-compatible expression vector plasmid (pOG44) coding for Flp recombinase using Lipofectamine 2000 (Thermo Fisher Scientific). For the selection of stably transfected cells, cells were cultured in DMEM containing 5% FCS supplemented with 15 μg/ml Blasticidin and 150 μg/ml Hygromycin (Invitrogen/Thermo Fisher Scientific). Expression of constructs was induced by the addition of 1 μg/ml doxycycline (Sigma-Aldrich).

### Clonogenic survival assays

For clonogenic survival assays, HeLa FIT cells and derivatives were seeded at 300 cells/well in a 24-well plate. 16h after *FANCJ* induction with 1 μg/ml doxycycline (Sigma-Aldrich), the cells were treated for 24h with different concentrations of mitomycin C (Sigma-Aldrich) or the G4-stabilising agents PDS (Tocris) and CX-5461 (Adipogen). After the treatment, the cells were washed with PBS and kept in standard growth medium. 8 days after seeding, the cells were washed with PBS, fixed with 100% methanol and incubated in staining solution (0.5% Crystal Violet, 25% methanol) for 5 min. Residual staining was removed with tap water and the 24-well plates were air-dried prior to scanning. Using the ColonyArea ImageJ plug-in [47], signal intensity and colony area were analysed.

### Whole cell extracts and immunoprecipitation experiments

Whole cell extracts (WCE) were prepared using 3 packed cell volumes (PCVs) of buffer C containing 0.1% Benzonase (Santa Cruz) and protease inhibitors (Roche). Lysates were kept on ice for 30 minutes and spun for 30 minutes at 17’000 x g and 4°C.

### Western blotting

Western blotting was performed using a standard protocol. Briefly, protein samples were boiled 5 minutes at 95°C in sample buffer and separated on a 10% SDS-PAGE for 1h at 180 V. Proteins were transferred to a nitrocellulose membrane for 1h at 100V using a wet transfer system. The membrane was then blocked for 30 minutes in 5% milk in PBS-T (50 mM Tris-HCl (pH 7.5), 150 mM NaCl and 0.01% Tween 20) and incubated in the primary antibody in a 1/1000 dilution in 5% milk/PBS-T overnight. The following day, membranes were washed in PBS-T and incubated in the secondary antibody (1/5000 in 5% milk/PBS-T) for 1h at room temperature (RT). Afterwards, the membrane was washed again extensively in PBS-T and developed with Clarity Western ECL Blotting Substrate (Bio-Rad) or SuperSignal West Femto Maximum Sensitivity Substrate (Thermo Scientific).

### Primary and secondary antibodies

Antibodies used in this study are listed in S6 Table.

### SMLM sample preparation and imaging

HeLa FIT cells and derivatives were trypsinised and seeded on glass coverslips (Fisher Scientific, 12-548-B) in six-well plates at low density and allowed to attach. Expression of *FANCJ* was induced with 1 μg/ml doxycycline for 23h. Cells were then treated for 1h with 20 μM PDS (Sigma, SML0678) before fixation. For nascent DNA detection, cells were treated with 10 μM EdU for 15 minutes before fixation, to make sure that EdU only incorporates into newly-synthesised DNA through endogenous replication. An optimised permeabilisation and fixation protocol was used to remove the majority of the cytoplasm and non-chromatin bound proteins in order to minimise nonspecific antibody labelling, which could significantly contribute to the noise for image analysis. Briefly, cells were permeabilised with 0.5% Triton X-100 in ice-cold CSK buffer (10 mM Hepes, 300 mM Sucrose, 100mM NaCl, 3mM MgCl_2_, pH 7.4) for 10 minutes at room temperature. Following pre-extraction, cells were washed once with PBS, then fixed in 3.7% paraformaldehyde (Electron Microscopy Sciences, 15714) in PBS for 30 minutes at room temperature. Cells were then washed twice with PBS and blocked with blocking buffer (2% glycine, 2% BSA, 0.2% gelatine, and 50 mM NH_4_Cl in PBS) at least overnight at 4°C prior to immunofluorescence staining and imaging.

Incorporated EdU was labelled using the Click-iT Plus EdU Alexa Fluor 647 Imaging Kit (ThermoFisher, C10640). DNA G4, MCM, and PCNA were labelled either directly by Alexa Fluor (AF)-conjugated primary antibodies in blocking buffer for 1h, or indirectly using primary antibodies for 1h, then Alexa Fluor secondary antibodies for 30 minutes. All staining steps were done at room temperature.

After immunofluorescence staining, coverslips with fixed cells were mounted on microscope glass slides with freshly prepared SR imaging buffer (1 mg/mL glucose oxidase (Sigma, G2133), 0.02 mg/mL catalase (Sigma, C3155), 10% glucose (Sigma, G8270), 100 mM mercaptoethylamine (Fisher Scientific, BP2664100) in PBS, pH 8.0) flowed through.

All raw SMLM-SR images were acquired using a custom-built optical imaging platform based on a Leica DMI 300 inverse microscope. 750 nm (UltraLaser, MDL-III-750-500), 639 nm (UltraLaser, MRL-FN-639-800), 561 nm (Cobolt), 488 nm (OBIS) laser lines were adjusted to 1.5, 0.8, 1.0, 0.8 kW/cm2, respectively. The laser lines were combined using appropriate dichroic and focused onto the back aperture of an HCX PL APO 63X NA = 1.47 OIL CORR TIRF (Zeiss) Objective *via* a multi-band dichroic (FF408/504/581/667/762-Di01-22x29). To increase power density and limit out-of-plane fluorescence, a Highly Inclined and Laminated Optical (HILO) illumination configuration was achieved by translating the excitation beam laterally across the back aperture of the objective. Fluorescence emission was expanded with a 2X lens tube, corrected by a chromatic aberration correction lens (Thorlabs, AC254-300-A), and was collected on a sCMOS camera (Photometrics, Prime 95B). Fluorescence signals were collected sequentially using the corresponding single-band pass filters in a filter wheel (ThorLabs, FW102C): AF750 (Semrock, FF02-809/81), AF488 (Semrock, FF01-531/40), AF647 (Semrock, FF01-676/37), AF568 (Semrock, FF01-607/36). A 405 nm laser line (MDL-III-405-150, CNI) was introduced to enhance recovery of dark state fluorophores when required. 2000 Frames at 33 Hz were acquired for each colour.

Mapping among different channels for multi-colour imaging was carried out using a polynomial morph-type mapping algorithm in order to correct the chromatic aberrations caused by the varying diffraction behaviours of different wavelength emissions [29]. Before each experiment, a calibration map was generated by imaging spatially separated fluorescent beads (ThermoFisher, T-7279) in each of the four channels. A 2nd polynomial function was optimised to fit the localizations of the beads in each of the AF750, AF568, and AF488 channels to their locations in the AF647 channel. This optimised 2nd polynomial function was then used to map the molecular localizations of the experimental samples in each of AF750, AF568, AF488 channels to the AF647 channel.

### Single-molecule localisation

Each frame of the raw image stack was firstly box-filtered with a box size of 4 times of the FWHM of a 2D Gaussian PSF. Note that each pixel of the image was weighted by the inverse of its pre-calibrated variance during the box-filtering [48]. The low-pass filtered image was then extracted from the raw image for rough local maxima recognition and localization. All the 7x7 pixel regions around all the local maxima from all frames of the image stack were then submitted for 2D-Gaussian multi-PSF fitting [49], which is performed by GPU (Nvidia GTX 1060, CUDA 8.0) using the Maximum Likelihood Estimation (MLE) algorithm. In brief, the likelihood function of each pixel was built by the convolution of 1) the Poisson distribution of the shot noise from the photons emitted from fluorophores nearby and 2) the gaussian distribution of the inherent read-out noise of each pixel pre-calibrated as mentioned above. The fitting accuracy was then estimated by Cramér-Rao lower bound (CRLB), and the distribution of the accuracy of all sequential localizations were fitted into a skew-Gaussian distribution. Any localizations appearing in consecutive frames within 2.5 times of the localization precision were considered as one blinking event. Such localizations were weighted by the inverse of its own CRLB determined variance and averaged into one localization in order to minimize overcounting during Auto-PC computation. For display purpose, the representative images were generated by rendering the raw coordinates into 10 nm pixel canvas and convolved with a 2D-Gaussian (σ = 10 nm) kernel.

### Triple-correlation Function (TCF)

Details of the TCF were described previously [29,50]. Briefly, the TCF is defined as Eq (1),

$$f(r_{1},r_{2})=\frac{{\langle \delta \rho_{1}(R)\delta \rho_{2}(R+r_{1})\delta \rho_{3}(R+r_{2})\rangle }_{R}}{{\langle \rho_{1}(R)\rangle }_{R}{\langle \rho_{2}(R)\rangle }_{R}{\langle \rho_{3}(R)\rangle }_{R}}$$ (1)

where 〈*ρ*_*i*_(**R**)〉_**R**_ denotes the average density of the detections from the *i*th of the three-color channels within the Region-Of-Interests (ROI, a ~6×6 μm^2^ square at the center of the 3-color SMLM image of a nucleus) and *δρ*_*i*_(**R**) = *ρ*_*i*_(**R**) − 〈*ρ*_*i*_(**R**)〉_**R**_ denotes the local density fluctuation at **R**.

### Estimation of the local density within a TC triplet pattern via TCF

〈*δρ*_1_(**R**)*δρ*_2_(**R** + **r**_**1**_)*δρ*_3_(**R** + **r**_**2**_)〉_**R**_ defines, on average, the product of the local density of the three species within a triplet pattern Δ(**r**_**1**_, **r**_**2**_), while 〈*δρ*_1_(**R**)*δρ*_2_(**R** + **r**_**1**_)〉_**R**_ stands for the average product of the two species correlating at **r**_**1**_. Similar to the conditional probability, the local density of the third species within the triple-pattern is therefore estimated as the ‘conditional’ local density at **r**_**2**_ − **r**_**1**_ given a pair correlating at **r**_**1**_ (Eq (2)):

$$C_{3}(r_{1},r_{2})=\frac{{\langle \delta \rho_{1}(R)\delta \rho_{2}(R+r_{1})\delta \rho_{3}(R+r_{2})\rangle }_{R}}{{\langle \delta \rho_{1}(R)\delta \rho_{2}(R+r_{1})\rangle }_{R}}$$ (2)

### Computation of TCF

Since SMLM data consists of coordinates other than intensity values at each pixel across the entire image canvas, we directly calculated the TCF as its definition (Eq (1)) by visiting each coordinate in the first channel, and calculated *δρ*_2_(**r**_**1**_) and *δρ*_3_(**r**_**2**_) in the second and third channels at **r**_**1**_, and **r**_**2**_ displaced from the visited coordinate, respectively. Moreover, since the triplets are randomly oriented in the ROI, the TCF at **r**_**1**_ = (*r*_1_, *θ*), **r**_**2**_ = (*r*_2_, *θ* + Δ*θ*) was averaged along *θ* ∈ [−*π*, *π*], and *f*(**r**_**1**_, **r**_**2**_) was thus transformed to *f*(*r*_1_, *r*_2_, *r*_3_) where $r_{3}^{2}=r_{1}^{2}+r_{2}^{2}+2r_{1}r_{2}\text{cos}\;\Delta \theta$.

## Supporting information

S1 Fig FANCJ coordinates an FeS cluster that is essential for MMC resistance. (A) Purified Flag-FANCJ variants from one representative iron-55 incorporation assay, as analysed by SDS-PAGE and InstantBlue staining. Protein amounts were taken into consideration for quantification. (B) Representative Western blot of *FANCJ* knock-out cells (FANCJ^–/–^) complemented with *FANCJ* variants. Expression of variants was induced by addition of 1 μg/ml doxycycline for 24h. Note that a non-specific band in the knock-out cell line, which is not FANCJ, runs at a similar level as FANCJ. P, parental HeLa FIT cell line; end., endogenous; WT, wild-type; KR, K52R; RQ, R279Q; CS, C283S; CH, C283H; CR, C283R; MI, M299I; LF, L340F; AP, A349P.
(TIF)

S2 Fig FeS cluster loss affects DNA unwinding, but not ATP hydrolysis and DNA binding. (A) ATP hydrolysis by 70 nM of FANCJ variants in the absence of DNA (no) and in the presence of oligonucleotide-based Y-structure DNA (Y) or D-loop substrates (D) was analysed by thin layer chromatography. (B) EMSAs showing increasing amounts (10/40/70 nM) of FANCJ variants incubated with an oligonucleotide-based Y-structure substrate. (C) DNA helicase assays showing DNA unwinding of an oligonucleotide-based D-loop substrate incubated with increasing amounts (10/40/70 nM) of FANCJ variants. 95, boiled sample; Pi, inorganic phosphate; WT, wild-type; KR, K52R; CS, C283S; CR, C283R; MI, M299I; LF, L340F; AP, A349P.
(TIF)

S1 Table Raw data and statistical analysis of clonogenic survival assays with MMC. Raw values and statistical analysis of MMC sensitivity (Fig 1C). Mean values (mean) and standard deviations (SD) from three independent experiments are shown. Ordinary Two-Way ANOVA was used for multiple comparisons (****, *p* < 0.0001; ***, *p* < 0.001; **, *p* < 0.01; *, *p* < 0.1; ns, non-significant). HeLa FIT, parental HeLa FIT cell line; FJ^–/–^, *FANCJ* knock-out cell line; WT, wild-type; KR, K52R; RQ, R279Q; CS, C283S; CH, C283H; CR, C283R; MI, M299I; LF, L340F; AP, A349P.
(XLSX)

S2 Table Raw data and statistics of SMLM analysis of G4 structures. Raw values and statistical analysis of densities of replisome-associated G4 structures in *FANCJ* knock-out cells complemented with the indicated variants (Fig 4B and 4C). Unpaired two-sample t-tests between NT and PDS-treated cells were used to determine *p*-values. KO, *FANCJ* knock-out cell line; WT, wild-type; KR, K52R; CS, C283S; CR, C283R; MI, M299I; LF, L340F; AP, A349P.
(XLSX)

S3 Table Raw data and statistical analysis of clonogenic survival assays with PDS. Statistical analysis of PDS sensitivity (Fig 4D). Mean values (mean) and standard deviations (SD) from three independent experiments are shown. Ordinary Two-Way ANOVA was used for multiple comparisons (**, *p* < 0.01; *, *p* < 0.1; ns, non-significant). FJ^–/–^, *FANCJ* knock-out cell line; WT, wild-type; KR, K52R; CS, C283S; CR, C283R; MI, M299I; LF, L340F; AP, A349P.
(XLSX)

S4 Table Raw data and statistical analysis of clonogenic survival assays with CX-5461. Statistical analysis of CX-5461 sensitivity (Fig 5A). Mean values (mean) and standard deviations (SD) from three independent experiments are shown. Ordinary Two-Way ANOVA was used for multiple comparisons (****, *p* < 0.0001; **, *p* < 0.01; *, *p* < 0.1; ns, non-significant). HeLa FIT, parental HeLa FIT cell line; FJ^–/–^, *FANCJ* knock-out cell line; WT, wild-type; KR, K52R; CS, C283S; CR, C283R; MI, M299I; LF, L340F; AP, A349P.
(XLSX)

S5 Table Oligonucleotide-based DNA substrates. FAM denotes fluorescein amidite label.
(DOCX)

S6 Table Antibodies used in this study. (DOCX)
