# Supplementary figures and images for: Cancer-associated mutations in the iron-sulfur domain of FANCJ affect G-quadruplex metabolism
Source: PLoS Genet. 2020 Jun 15;16(6):e1008740. doi: 10.1371/journal.pgen.1008740 (PMC7316351; doi:10.1371/journal.pgen.1008740)

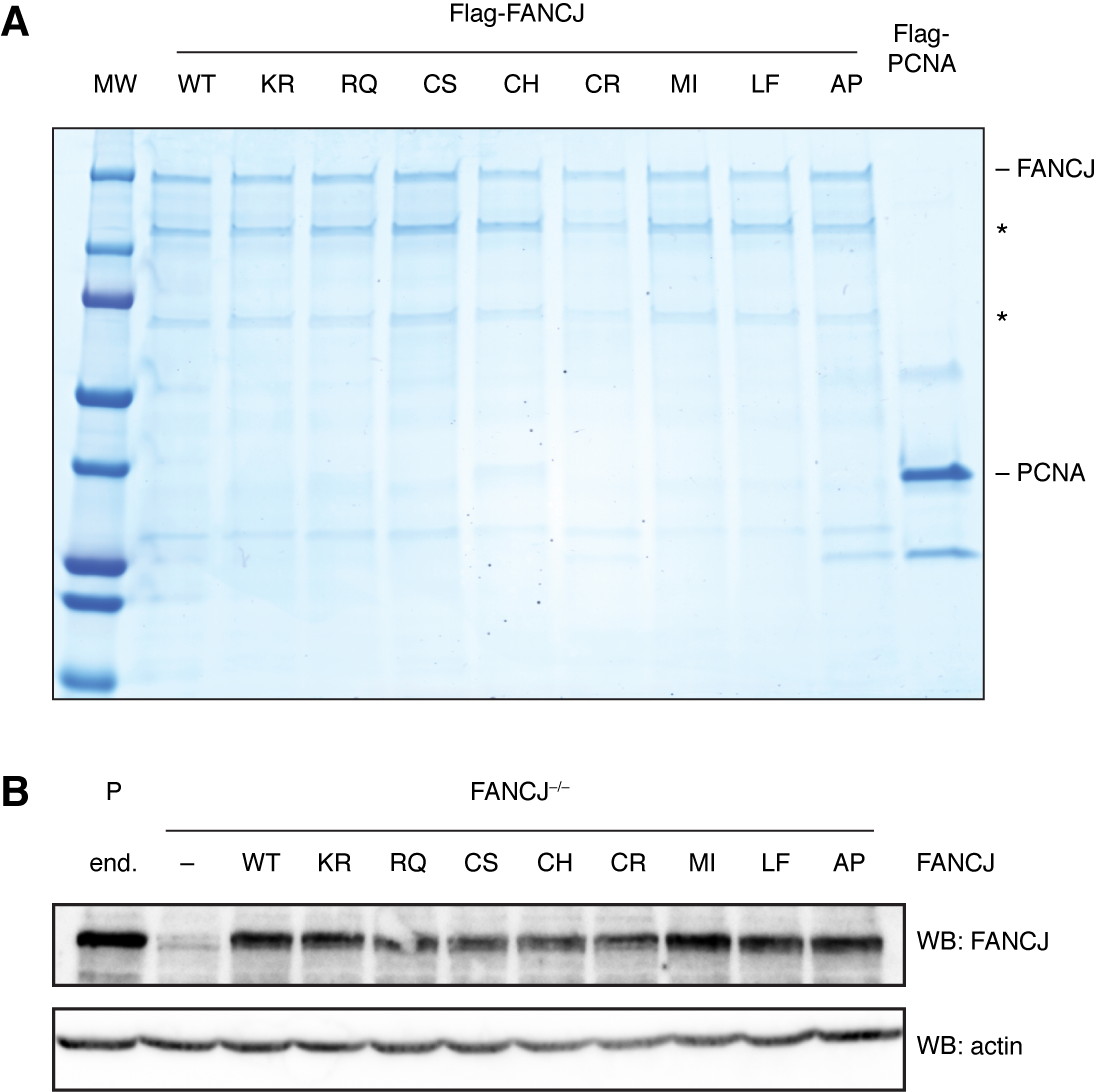

Supplement: S1 Fig — (A) Purified Flag-FANCJ variants from one representative iron-55 incorporation assay, as analysed by SDS-PAGE and InstantBlue staining. Protein amounts were taken into consideration for quantification. (B) Representative Western blot of FANCJ knock-out cells (FANCJ–/–) complemented with FANCJ variants. Expression of variants was induced by addition of 1 μg/ml doxycycline for 24h. Note that a non-specific band in the knock-out cell line, which is not FANCJ, runs at a similar level as FANCJ. P, parental HeLa FIT cell line; end., endogenous; WT, wild-type; KR, K52R; RQ, R279Q; CS, C283S; CH, C283H; CR, C283R; MI, M299I; LF, L340F; AP, A349P. (TIF) [file pgen.1008740.s001.tif]

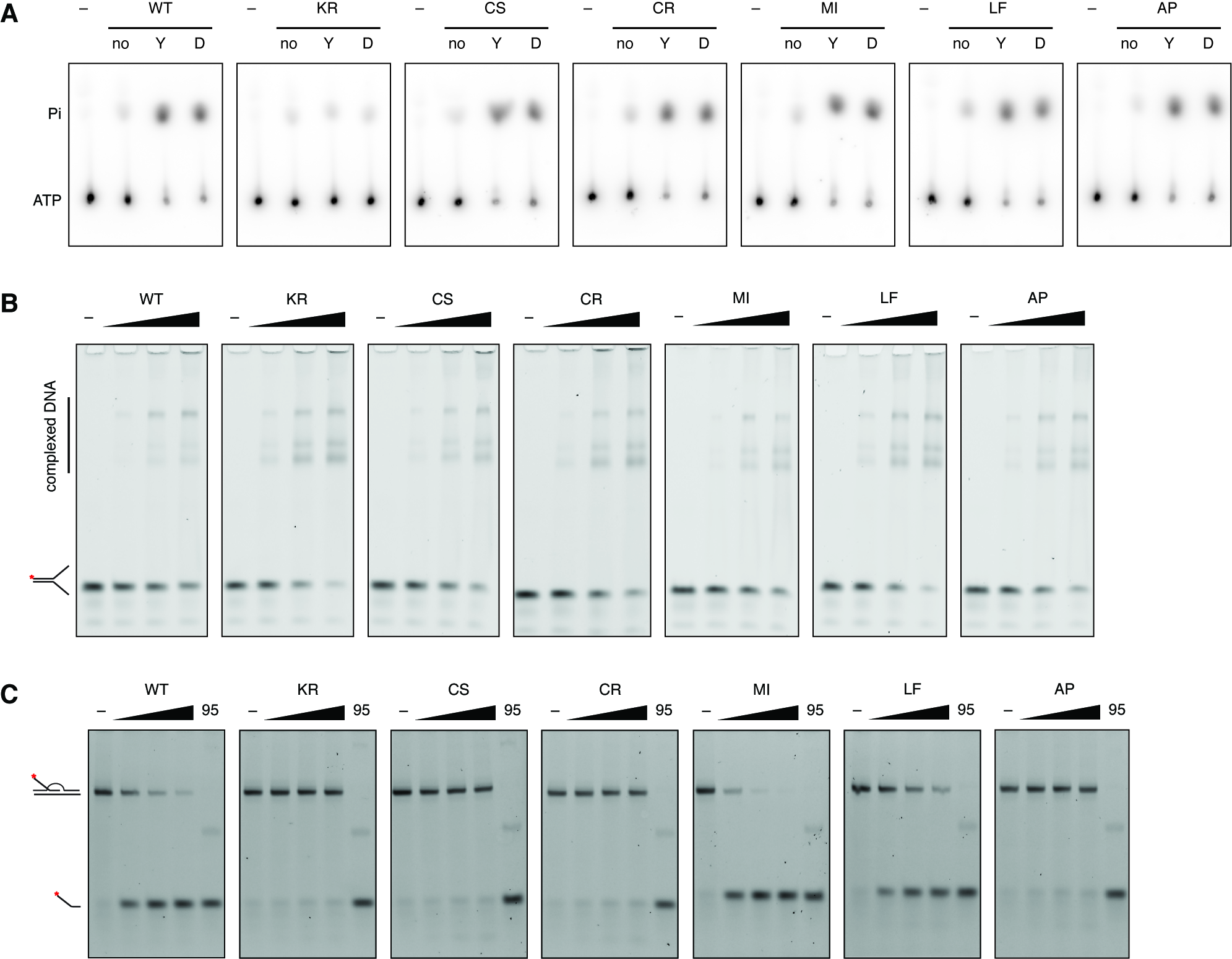

Supplement: S2 Fig — (A) ATP hydrolysis by 70 nM of FANCJ variants in the absence of DNA (no) and in the presence of oligonucleotide-based Y-structure DNA (Y) or D-loop substrates (D) was analysed by thin layer chromatography. (B) EMSAs showing increasing amounts (10/40/70 nM) of FANCJ variants incubated with an oligonucleotide-based Y-structure substrate. (C) DNA helicase assays showing DNA unwinding of an oligonucleotide-based D-loop substrate incubated with increasing amounts (10/40/70 nM) of FANCJ variants. 95, boiled sample; Pi, inorganic phosphate; WT, wild-type; KR, K52R; CS, C283S; CR, C283R; MI, M299I; LF, L340F; AP, A349P. (TIF) [file pgen.1008740.s002.tif]
