# Supplementary material for: Cancer-associated mutations in the iron-sulfur domain of FANCJ affect G-quadruplex metabolism
Source: PLoS Genet. 2020 Jun 15;16(6):e1008740. doi: 10.1371/journal.pgen.1008740 (PMC7316351; doi:10.1371/journal.pgen.1008740)
Supplement: S5 Table — FAM denotes fluorescein amidite label. (DOCX) [file pgen.1008740.s007.docx]

| **Substrate** | **Oligos** | **Sequence (5´–3´)** |
| --- | --- | --- |
| Y-structure | XO1 | 5´-FAM–GACGCTGCCGAATTCTACCAGTGCCTTGCTAGGACATCTTTG |
|  | sDO1 | TTTTTTTTTTTTTTTTTTTTTTTTTTTTTTTTTTTTGGTAGAATTCGGCAGCGT |
| D-loop | invading strand | 5´-FAM–TTTTTTTTTTTTTTTTTTTTGGTTAGGGTTAGGGTTAACG |
|  | top strand | ATCACATACGCTTTGCTATTCCGGTTTTTTTTTTTTTTTTTTTTCCGTGCCACGTTGTATGCCCACGT |
|  | bottom strand | ACGTGGGCATACAACGTGGCACGGCGTTAACCCTAACCCTAACCCCGGAATAGCAAAGCGTATGTGAT |
|  | competitor DNA | GGTTAGGGTTAGGGTTAACG |
| Primer-template substrate with parallel G4 structure on template | template strand | GCGAACTTGAATTCTATTTTGGGTGGGTGGGTGGGTTTTCACATATGCAAAGATGTCCTAGCAAGGCACTGGTAGAATTCGGCAGCGTC |
|  | primer | 5´-FAM–  GACGCTGCCGAATTCTACCAGTGCCTTGCTAGGACATC TTTG |
|  | competitor DNA | GACGCTGCCGAATTCTACCAGTGCCTTGCTAGGACATCTTTGCATATGTGAAAACCCACCCACCCACCCAAAATAGAATTCAAGTTCGC |
