# Supplementary material for: Cancer-associated mutations in the iron-sulfur domain of FANCJ affect G-quadruplex metabolism
Source: PLoS Genet. 2020 Jun 15;16(6):e1008740. doi: 10.1371/journal.pgen.1008740 (PMC7316351; doi:10.1371/journal.pgen.1008740)
Supplement: S6 Table — (DOCX) [file pgen.1008740.s008.docx]

| **Target** | **Species** | **Provider** | **cat #** | **Application** | **Dilutions** |
| --- | --- | --- | --- | --- | --- |
| FANCJ | rabbit | Novus Biologicals | NBP-31883 | WB | 1:1000 |
| β-actin (C4), HRP-linked | mouse | Santa Cruz | sc-47778 | WB | 1:1000 |
| Rabbit IgG, HRP-linked | donkey | GE Healthcare | NA934 | WB | 1:5000 |
| DNA G4 (1H6) | rabbit | Absolute Antibody | Ab00389-23.0 | IF | 1:200 |
| PCNA | mouse | Santa Cruz | sc-56 | IF | 1:1000 |
| MCM6 – AF 568 | rabbit | Abcam | ab211916 | IF | 1:1000 |
| Rabbit IgG ­– AF 750 | goat | Invitrogen | A21039 | IF | 1:5000 |
| Mouse IgG – AF 488 | goat | Invitrogen | A11029 | IF | 1:5000 |
